# Phytate Decreases Formation of Advanced Glycation End-Products in Patients with Type II Diabetes: Randomized Crossover Trial

**DOI:** 10.1038/s41598-018-27853-9

**Published:** 2018-06-25

**Authors:** Pilar Sanchis, Rosmeri Rivera, Francisco Berga, Regina Fortuny, Miquel Adrover, Antonia Costa-Bauza, Felix Grases, Luis Masmiquel

**Affiliations:** 1 Endocrinology Department, Research Unit, Hospital Son Llàtzer, Institute of Health Sciences Research [IUNICS- IdISBa], 07198 Palma of Mallorca, Spain; 2 Laboratory of Renal Lithiasis Research, University of Balearic Islands, Institute of Health Sciences Research [IUNICS- IdISBa], 07122 Palma of Mallorca, Spain; 3 Laboratory Department, Hospital Son Llàtzer, 07198 Palma of Mallorca, Spain; 4 Department of Chemistry, University of Balearic Islands, Ctra. Valldemossa km 7.5, 07122 Palma of Mallorca, Spain

## Abstract

Myo-inositol hexaphosphate (phytate; IP6) is a natural compound that is abundant in cereals, legumes, and nuts and it has the ability to chelate metal cations. The binding of IP6 to transition metals suggests that it could be used for the treatment of metal-catalyzed protein glycation, which appears to trigger diabetes-related diseases. Our *in vitro* studies showed that IP6 reduced the formation of Fe^3+^-catalyzed advanced glycation end-products (AGEs). This led us to perform a randomized cross-over trial to investigate the impact of the daily consumption IP6 on protein glycation in patients with type 2 diabetes mellitus (T2DM; n = 33). Thus, we measured AGEs, glycated hemoglobin (HbA1c), several vascular risk factors, and urinary IP6 at baseline and at the end of the intervention period. Patients who consumed IP6 supplements for 3 months had lower levels of circulating AGEs and HbA1c than those who did not consume IP6. This is the first report to show that consumption of IP6 inhibits protein glycation in patients with T2DM. Considering that AGEs contribute to microvascular and macrovascular complications in T2DM, our data indicates that dietary supplementation with IP6 should be considered as a therapy to prevent the formation of AGEs and therefore, the development of diabetes-related diseases in patients with T2DM.

## Introduction

Type 2 diabetes mellitus (T2DM) is a common endocrine disorder, in which chronic insulin resistance and progressive failure of β-cells leads to hyperglycemia^1^. Worldwide, 382 million people are currently diagnosed of T2DM, and this number is expected to reach 600 million in 2035^2^.

Hyperglycemia induces the development of diabetes-related disorders, such as nephropathy^3^, retinopathy^4^, hypertension^5^ or dyslipidemia^6^. Moreover, recent studies reported positive correlations of T2DM with Alzheimer’s disease^7,8^ and with Parkinson’s disease^9,10^, although the detailed molecular mechanisms that follow from hyperglycemia to these diseases are uncertain. However, one of the main triggering factors seems to be protein glycation and the consequent formation of advanced glycation end-products (AGEs)^11,12^. It is assumed that the accumulation of AGEs modifies the protein structure causing loss-of-function, which may lead to development of diabetes-related diseases^13,14^. However, we recently demonstrated that protein glycation does not necessary involve structural modifications^15,16^, in agreement with other contributions^17,18^.

Protein glycation starts with the reaction between reducing sugars and the protein Lys side chains, a process enhanced in patients with T2DM due to their increased levels of serum glucose (>7 mM;^19^). This reaction initially yields a Schiff base that rearranges into an Amadori product, which is the key factor that enables the final formation of AGEs through a metal-catalyzed mechanism. AGEs are a heterogeneous family of compounds with different chemical features, which are mainly formed on protein Lys, Arg, and Cys side chains. Hence, their chemical structure depends on the glycating compound, the protein target, the chemical environment of each glycation hot-spot, and other factors. Therefore, a diverse set of AGEs can be formed *in vivo* on a single amino-acid residue (non-crosslinking AGEs) and/or between different amino-acid residues (crosslinking AGEs)^20–22^.

Several mechanisms may explain the relationship between AGEs and diabetes complications: *(i)* accumulation of AGEs in the extracellular matrix, causing crosslinking and decreased vessel elasticity (increased arterial stiffness);^23^ *(ii)* binding to specific AGEs receptors (RAGEs) that activate certain cell signaling pathways, such as NF-κB pathway, and modulate gene expression in different cell types;^24^ *(iii)* alteration of protein structure and function;^25^ and *(iv)* quenching of intracellular nitric oxide and disrupting the function of certain growth factors^26^.

The role of AGEs in the development of diabetes-related diseases has focused therapeutic investigations towards the search of compounds that can inhibit protein glycation. Several pharmacological approaches can inhibit AGEs formation: *(i)* trapping of reactive carbonyl compounds; *(ii)* chelation of metal cations that are important in the catalysis of AGEs formation; *(iii)* blocking of RAGEs; *(iv)* scavenging of free radicals formed during protein glycation; *(v)* cleavage of crosslinking AGEs; and *(vi)* reducing serum glucose^27^. Thus, several compounds (*e*.*g*. pyridoxamine, benfotiamine, aminoguanidine, ALT-711, *etc*.) have potential as anti-glycation agents because they act through one or several of these mechanisms^27,28^. Among them, pyridoxamine stands out because it scavenges carbonyl compounds, traps reactive oxygen species, and inhibits oxalate biosynthesis^29^. Pyridoxamine has also the ability to strongly chelate metal cations^30^, thus inhibit the formation of AGEs directly arising from Amadori compounds^31^. Because metal-catalyzed reactions play a major role in the formation of AGEs^32–35^, a therapeutic approach based on chelation of metal cations, such as Cu^2+^ or Fe^3+^, has emerged as potential therapy against protein glycation.

Myo-inositol hexaphosphate (phytate, IP6) is a natural compound present in seeds (*e*.*g*. cereals, legumes, and nuts) as a calcium-magnesium salt (phytin)^36^, that can chelate most divalent cations^37,38^. IP6 has also a high affinity towards Fe^3+^, being even greater than that of EDTA, deferoxamine (DFOA) or ATP^39^, thus suggesting that IP6 consumption could reduce mineral bioavailability^40,41^. Nevertheless, several studies have indicated that dietary phytin intake of 2 g/day, as part of a balanced diet, had no adverse effects on mineral bioavailability^42–46^. In addition, we have also shown that IP6 prevents the formation of pathological calcifications *in vivo*, such as renal calculi^47,48^, dental calculi^49^, and cardiovascular calcification^50–52^, and protects against osteoporosis^53^. Moreover, IP6 may also provide protection against cancer^54^ and Parkinson’s disease^55^.

The remarkable ability of IP6 to chelate Fe^3+^, together with the potential of this cation as a catalyst of the AGEs formation processes^56–58^, motivated us to study whether IP6 could act as inhibitor of the metal-catalyzed glycation. Thus, the specific aims of this paper are: *(i)* study the ability of IP6 to inhibit protein glycation *in vitro*; *(ii)* and investigate the impact of daily IP6 consumption on metabolic and glycemic control, and on the *in vivo* formation of AGEs in patients with T2DM.

## Results

### Effect of IP6 on the formation of AGEs *in vitro*

**Figure 1:**
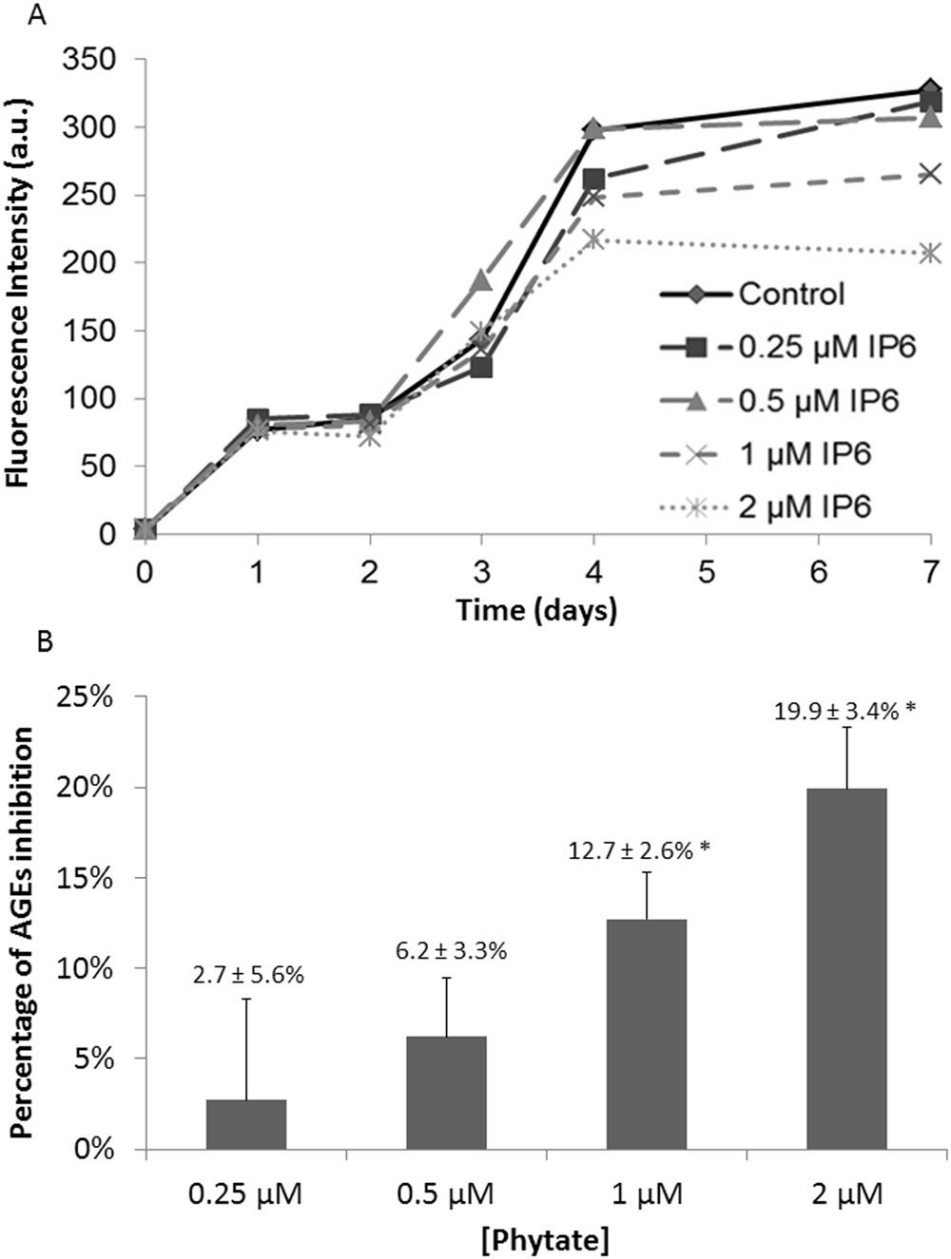
Effect of IP6 concentration on *in vitro* formation of AGEs. (**A**) Changes of fluorescence intensity signal (λ_exc_ 320 nm; λ_em_ 420 nm) a different incubation times of a solution containing Ac-Lys (2 mM), Ac-Arg (1 mM), ribose (0.2 M), and 2 µM Fe^3+^ with 0 to 2 µM IP6. (**B**) Percentage inhibition of AGEs formation by different concentrations of IP6 after 7 days of incubation *p < 0.05 *vs*. control (0 µM).

IP6 is a powerful Fe^3+^ chelator^39^, so we hypothesized that it could block the steps along the glycation process that are sensitive to Fe^3+^ catalysis. Thus, we initially used fluorescence spectroscopy to measure the effect of IP6 concentration (0 to 2 µM) on the time-dependent formation of Fe^3+^-catalyzed AGEs. These studies were carried out on a solution containing N^α^-acetyl-lysine (Ac-Lys), N^α^-acetyl-arginine (Ac-Arg), ribose and Fe^3+^, which mimicked a protein glycation process that could occur *in vivo*. The obtained results showed that the fluorescent signal temporally increased over 7 days due to the formation of ribose-derived fluorescent AGEs (Fig. 1A), as we previously described^15^. Although IP6 concentrations of 0.5 µM and below had no effect on the AGEs formation, the fluorescence signal increase was lower in the presence of 1 and 2 µM IP6. However, the inhibitory effects of IP6 at these concentrations were only significant on day-4 and day-7 (Fig. 1B), thus suggesting that IP6 may inhibit late steps in the glycation pathway, such as post-Amadori reactions.

### Baseline characteristics of patients

**Figure 2:**
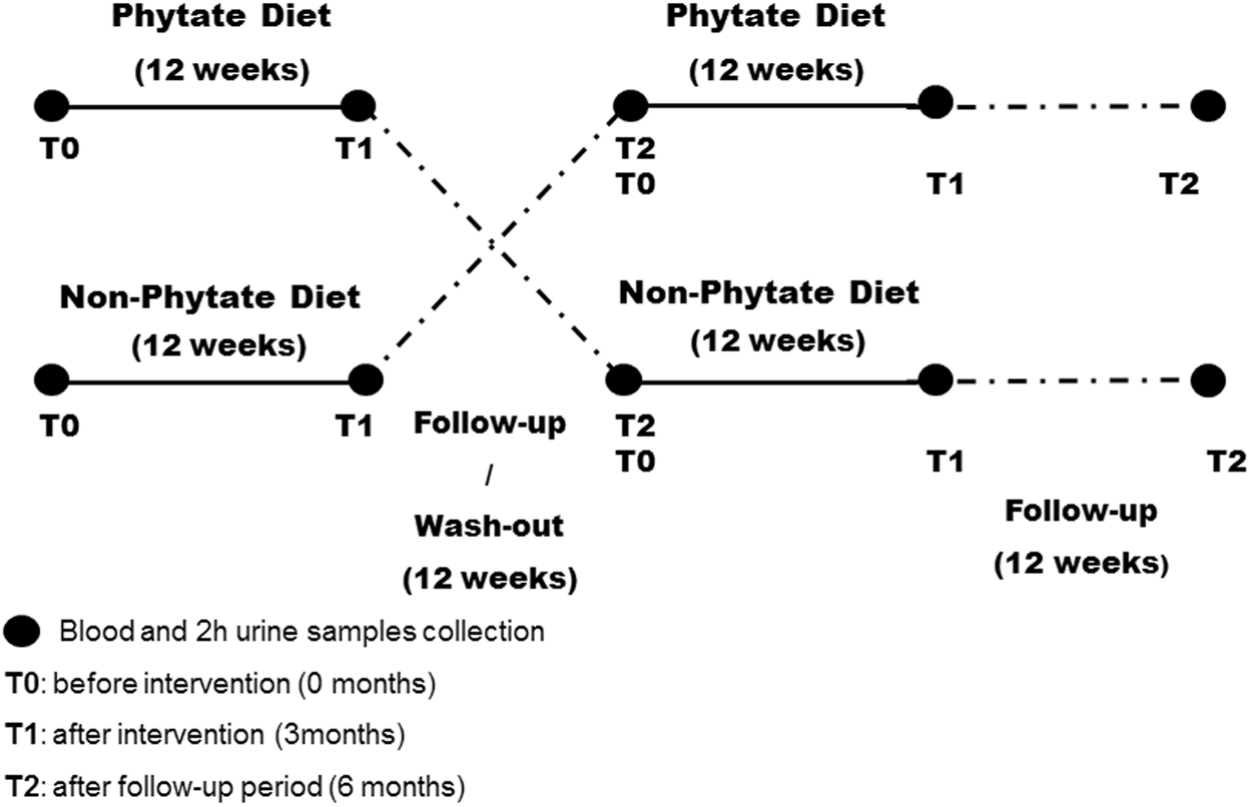
Design of the randomized crossover study of patients with T2DM (n = 33).

*In vitro* data indicating an inhibitory effect of IP6 on fluorescent AGEs formation, led us to design an interventional randomized crossover trial study to analyze whether these positive results would also be observed on T2DM patients consuming a diet rich in IP6 during 12 weeks **(**Fig. 2**)**. Thirty-three patients (20 females and 13 males) completed the clinical study **(**Table 1**)**. The median age was 64 years (interquartile range [IQR]: 52 to 70) and the mean duration of T2DM was 11 years (IQR: 6 to 14 years). Regarding dietary phytate consumption at baseline, the median of estimated intake was 260 mg/day (IQR: 150 to 426 mg/day). As can be expected, it was very low when compared with the IP6 consumption in a typical Mediterranean diet (1 g/day)^59^.

**Table 1:** Baseline characteristics of patients (n = 33). Each value is given as median (interquartile range) or number (%).

Baseline characteristics (n = 33)		
Age (years)		64 (52–70)
Duration of DM (years)		11 (6–14)
Estimated phytate intake (mg/day)		260 (150–426)
Sex (female)		20 (60.6%)
DM complications		8 (24.2%)
Type of complications	Erectile dysfunction	2 (6.1%)
	CKD+ retinopathy	1 (3.0%)
	Neuropathy + retinopathy	2 (6.1%)
	Retinopathy	3 (9.1%)
**Comorbidities**		
Smoking (ex or yes)		4 (12.1%)
Alcohol (ex or yes)		5 (15.2%)
Dyslipidemia		20 (60.6%)
Hypertension		20 (60.6%)
**Medication use**		
Insulin		19 (57.6%)
Oral anti-diabetic drugs (OAD)		30 (90.9%)
N° OAD	0	3 (9.1%)
	1	12 (36.4%)
	2	14 (42.4%)
	3	4 (12.1%)
α-GLP-1		7 (21.2%)
Statins		26 (78.8%)
Fibrates		2 (6.1%)
Antihypertensives		20 (60.6%)
N° antihypertensive drugs	0	13 (39.4%)
	1	7 (21.2%)
	2	7 (21.2%)
	3	4 (12.1%)
	4	1 (3.0%)
	5	1 (3.0%)
Dyslipidemia treatment		23 (69.7%)
N° dyslipidemia drugs	0	9 (27.3%)
	1	21 (63.6%)
	2	3 (9.1%)

Five patients (15.2%) consumed alcohol, 4 patients smoked (12.1%), and 8 patients (24.2%) had complications associated with T2DM. Thirty patients (90.9%) were on oral antidiabetic agents, and 19 (57.6%) used insulin therapy. Twenty (60.6%) were on antihypertensive therapy and 23 (69.7%) were treated with lipid-lowering agents. During the study period, there were no changes in medication usage (Table 1). The anthropometric and laboratory analysis variables before starting both diets did not reveal differences between the two groups (Table 2).

**Table 2:** Anthropometric and laboratory values before starting each diet.

	Before phytate diet	Before non-phytate diet	Intergroup comparison *p*-value
	median (Q1–Q3)	median (Q1–Q3)	
Weight (kg)	88 (78–98)	87 (79–96)	0.768
BMI (kg/m^2^)	36 (33–41)	36 (33–40)	0.768
Waist (cm)	106 (103–116)	107 (102–114)	0.778
Hip (cm)	110 (102–115)	110 (103–116)	0.878
Waist/Hip	0.99 (0.95–1.01)	0.99 (0.96–1.02)	0.709
Glucose (mg/dL)	174 (136–197)	170 (129–209)	0.663
Total cholesterol (mg/dL)	163 (139–198)	181 (144–212)	0.397
LDL cholesterol (mg/dL)	89 (80–134)	107 (80–134)	0.390
HDL cholesterol (mg/dL)	41 (35–50)	39 (35–45)	0.292
Triglycerides (mg/dL)	128 (98–208)	150 (109–185)	0.621
Insulin (µUI/mL)	22 (11–41)	21 (13–43)	0.649
Lipoprotein A (mg/dL)	21 (4–79)	11 (4–58)	0.554
hs-CRP (mg/dL)	0.37 (0.20–0.70)	0.36 (0.20–0.57)	0.793
Iron (µg/dL)	70 (55–93)	79 (61–103)	0.210
Ferritin (ng/mL)	49 (22–127)	55 (20–107)	0.945
Transferrin (mg/dL)	263 (246–288)	261 (239–285)	0.328
Transferrin saturation index (%)	19 (16–25)	22 (17–27)	0.226
Vitamin B12 (pg/mL)	390 (228–508)	337 (265–473)	0.762
Folate (ng/mL)	8.0 (5.4–11.0)	7.0 (4.6–9.9)	0.405
Leukocytes (×10^9^/L)	8.0 (7.1–9.9)	8.0 (6.4–9.5)	0.449
Lymphocytes T1 (×10^9^/L)	2.6 (2.1–3.5)	2.4 (1.9–2.9)	0.346
Lymphocytes (%)	33 (27–38)	31 (29–39)	0.836
Platelets (×10^9^/L)	256 (231–298)	257 (221–301)	0.762

Each value is given as median (interquartile range). The significance of differences between groups (intergroup comparison) was determined using the Mann-Whitney U test or an independent samples *t*-test.

### Effect of dietary IP6 on AGE, HbA1c, and other variables

**Figure 3:**
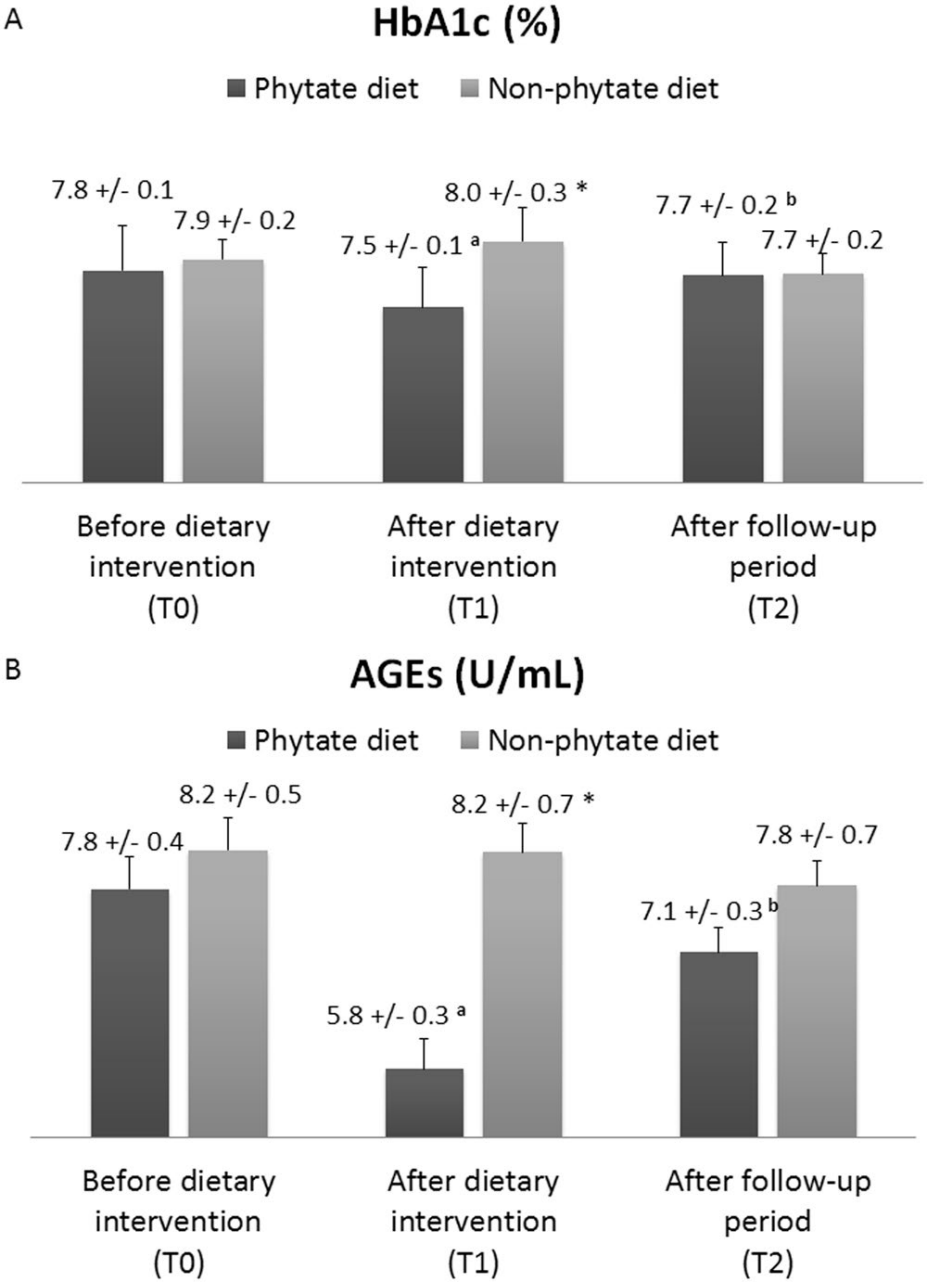
Effect of a phytate diet on serum levels of HbA1c (**A**) and AGEs (**B**). Values are expressed as means ± SEs. Intra-group differences (T_0_ *vs*. T_1_ and T_1_ *vs*. T_2_) were assessed using a paired-sample Wilcoxon signed-rank test or a paired-samples *t*-test (*a*: *p* < 0.05 for T_0_ *vs*. T_1_; *b: p* < 0.05 for T_1_ *vs*. T_2_). Inter-group differences (phytate diet *vs*. non-phytate diet) were assessed using an analysis of covariance after adjusting for baseline levels (*p < 0.05).

At baseline (T_0_, before intervention), the two groups had similar HbA1c and AGEs levels (Fig. 3). At 3 months (T_1_, after intervention), patients treated with the IP6 diet had significantly lowered their HbA1c (7.8 ± 0.1% to 7.5 ± 0.1%; *p* = 0.029) and AGEs (7.8 ± 0.4% to 5.8 ± 0.3%; *p* < 0.001) levels. At 6 months (T_2_, after follow-up period), patients who received the IP6 diet had significant increases in HbA1c and AGEs levels relative to T_1_ (HbA1c: 7.5 ± 0.1% to 7.7 ± 0.2%; *p* = 0.012; AGEs: 5.8 ± 0.3% to 7.1 ± 0.3%; *p* < 0.001). Patients given the non-IP6 diet had no significant changes in HbA1c and AGEs levels from T_0_ to T_2_.

The effect of IP6 intake on other risk factors was evaluated after the dietary intervention (T_1_ vs. T_0_; Table 3) and after follow-up period (T_2_ vs. T_1_; Table 4). No significant changes in the other clinical and biochemical parameters were detected. However, an unexpected but small increase in weight and BMI was observed at the end of the follow-up period (T_2_) in the IP6 dietary group **(**Table 4**)**. There was a minor (but not statistically significant) increase of total cholesterol detected after the IP6 dietary intervention (T_1_) with respect to the baseline (T_0_**)**. Also, a significant decrease in total cholesterol was observed after non-IP6 intervention **(**Table 3**)**. Regarding iron status, no deficit was found after IP6 dietary intervention. An slight increase in iron levels was observed after IP6 intervention (median change [IQ]: 4.0 (−0.25–16.0), *p* = 0.029). Nevertheless, no differences were observed in iron and transferrin levels between IP6 and non-IP6 intervention after adjusting for baseline values. No changes in inflammatory markers (CRP and leukocyte count) were observed.

**Table 3:** Changes in vascular risk factors after the diet intervention (T_1_, 3-months).

	After phytate diet (T_1_)		After non-phytate diet (T_1_)		Intergroup *p*-value
	median (Q1–Q3)	T_1_ vs. T_0_ *p*-value	median (Q1–Q3)	T_1_ vs. T_0_ *p*-value	
Weight (kg)	0.00 (−1.50–1.15)	0.658	0.00 (−2.60–2.10)	0.853	0.429
BMI (kg/m^2^)	0.00 (−0.62–0.48)	0.658	0.00 (−1.08–0.87)	0.853	0.429
Waist (cm)	0.00 (−0.01–0.00)	0.736	0.00 (0.00–0.01)	0.768	0.991
Hip (cm)	0.00 (0.00–0.00)	0.081	0.00 (−1.00–0.50)	0.739	0.150
Waist/Hip	0.00 (−0.01–0.00)	0.379	0.00 (0.00–0.01)	0.511	0.109
Glucose (mg/dL)	2.00 (−18.00–27.00)	0.454	−5.00 (−46.50–21.50)	0.396	0.345
Total cholesterol (mg/dL)	2.00 (−3.00–12.00)	0.060	−8.00 (−24.50–7.00)	0.019	0.005
LDL cholesterol (mg/dL)	3.60 (−6.90–8.40)	0.526	−4.20 (−16.40–6.50)	0.254	0.410
HDL cholesterol (mg/dL)	−1.00 (−4.50–3.00)	0.298	1.00 (−2.00–5.00)	0.243	0.156
Triglycerides (mg/dL)	9.00 (−38.00–47.00)	0.313	−11.00 (−39.50–8.50)	0.076	0.072
Insulin (µUI/mL)	−0.14 (−5.74–4.84)	0.688	−0.07 (−6.07–3.31)	0.823	0.181
Lipoprotein A (mg/dL)	0.00 (−4.37–2.20)	0.496	0.00 (−2.60–7.20)	0.356	0.513
hs-CRP (mg/dL)	−0.01 (−0.11–0.05)	0.422	0.03 (−0.02–0.20)	0.106	0.662
Iron (µg/dL)	4.00 (−0.25–16.00)	0.029	−4.00 (−19.00–8.50)	0.197	0.201
Ferritin (ng/mL)	2.74 (−9.51–9.87)	0.561	−0.86 (−16.19–6.11)	0.489	0.896
Transferrin (mg/dL)	0.00 (−18.50–9.50)	0.352	3.50 (−6.50–16.00)	0.252	0.919
Transferrin saturation index (%)	1.30 (−0.51–4.46)	0.048	−1.77 (−6.09–2.24)	0.172	0.531
Vitamin B12 (pg/mL)	−4.00 (−52.50–35.00)	0.326	−3.00 (−35.50–20.00)	0.550	0.137
Folate (ng/mL)	0.20 (−1.20–1.15)	0.925	−0.35 (−1.70–1.75)	0.852	0.253
Leukocytes (×10^9^/L)	−0.36 (−1.07–0.47)	0.292	−0.12 (−0.58–0.57)	0.837	0.158
Lymphocytes T1 (×10^9^/L)	−0.17 (−0.59–0.25)	0.198	0.00 (−0.24–0.35)	0.405	0.778
Lymphocytes (%)	−0.90 (−3.35–3.55)	0.501	0.70 (−2.80–2.05)	0.940	0.849
Platelets (×10^9^/L)	3.00 (−15.00–19.50)	0.75	5.00 (−8.50–21.00)	0.22	0.445

Un-adjusted within groups changes (before, T_0_ *vs*. after intervention, T_1_) are given as median (interquartile range). Intra-group analysis (T_0_ *vs*. T_1_) used a paired-sample Wilcoxon signed-rank test or paired-samples *t*-test to determine the significance of differences. Inter-group analysis (IP6 diet *vs*. non-IP6 diet) used analysis of covariances and comparison between groups after adjusting for baseline levels to determine the significance of differences.

**Table 4:** Changes in vascular risk factors after follow-up period (6 months).

	After follow-up phytate diet (T_2_)		After follow-up non-phytate diet (T_2_)		Intergroup *p*-value
	median (Q1–Q3)	T_1_ vs. T_2_ *p*-value	median (Q1–Q3)	T_1_ vs. T_2_ *p*-value	
Weight (kg)	0.70 (−0.45–1.85)	0.035	0.00 (−0.45–1.45)	0.156	0.920
BMI (kg/m^2^)	0.29 (−0.19–0.77)	0.035	0.00 (−0.19–0.60)	0.156	0.920
Waist (cm)	0.00 (0.00–0.00)	0.659	0.00 (0.00–0.00)	0.580	0.661
Hip (cm)	0.00 (0.00–0.00)	0.693	0.00 (0.00–1.00)	0.596	0.396
Waist/Hip	0.00 (0.00–0.00)	0.233	0.00 (0.00–0.00)	0.501	0.269
Glucose (mg/dL)	−1.00 (−21.00–27.00)	0.860	0.00 (−23.50–25.00)	0.974	0.734
Total cholesterol (mg/dL)	0.00 (−8.50–17.50)	0.352	0.00 (−8.00–13.00)	0.566	0.989
LDL cholesterol (mg/dL)	4.80 (−11.00–18.90)	0.074	0.80 (−8.00–4.45)	0.568	0.287
HDL cholesterol (mg/dL)	−1.00 (−3.00–1.00)	0.507	1.00 (−0.50–4.00)	0.043	0.104
Triglycerides (mg/dL)	−14.00 (−41.00–21.50)	0.175	5.00 (−7.50–22.50)	0.130	0.326
Insulin (µUI/mL)	1.23 (−2.79–6.50)	0.136	−0.59 (−2.53–4.12)	0.695	0.987
Lipoprotein A (mg/dL)	−0.40 (−4.44–0.50)	0.037	0.00 (−6.37–1.14)	0.561	0.427
hs-CRP (mg/dL)	0.00 (−0.14–0.06)	0.552	0.03 (−0.05–0.11)	0.453	0.059
Iron (µg/dL)	0.00 (−8.50–14.50)	0.611	0.00 (−14.00–13.00)	0.899	0.827
Ferritin (ng/mL)	−4.02 (−20.99–5.60)	0.289	−1.95 (−7.78–1.26)	0.055	0.033
Transferrin (mg/dL)	0.00 (−8.50–10.00)	0.888	−3.00 (−11.50–5.00)	0.094	0.300
Transferrin saturation index (%)	0.64 (−3.23–3.77)	0.453	0.00 (−3.76–3.72)	0.631	0.819
Vitamin B12 (pg/mL)	−14.00 (−49.00–35.50)	0.948	2.00 (−25.00–53.00)	0.196	0.280
Folate (ng/mL)	−1.00 (−2.95–0.25)	0.030	0.00 (−0.85–1.00)	0.344	0.736
Leukocytes (×10^9^/L)	−0.10 (−0.99–0.37)	0.171	0.03 (−1.00–0.57)	0.961	0.420
Lymphocytes T1 (×10^9^/L)	−0.07 (−0.24–0.09)	0.133	−0.05 (−0.22–0.24)	0.468	0.213
Lymphocytes (%)	0.10 (−2.10–2.55)	0.465	−0.60 (−3.70–2.80)	0.484	0.364
Platelets (×10^9^/L)	−1.0 (−21.0–13.0)	0.304	4.0 (−6.5–24.3)	0.048	0.451

Un-adjusted within groups changes (after dietary intervention, T_1_ *vs*. after follow-up period, T_2_) are given as median (interquartile range). Intra-group comparisons (T_1_ vs. T_2_) employed a paired-sample Wilcoxon signed rank test or paired-samples *t*-test to determine the significance of differences. Inter-group comparisons (IP6 diet *vs*. non-IP6 diet) employed an analysis of covariances after adjusting for dietary intervention levels to determine the significance of differences.

### Effect of dietary IP6 on urinary excretion of IPs

**Figure 4:**
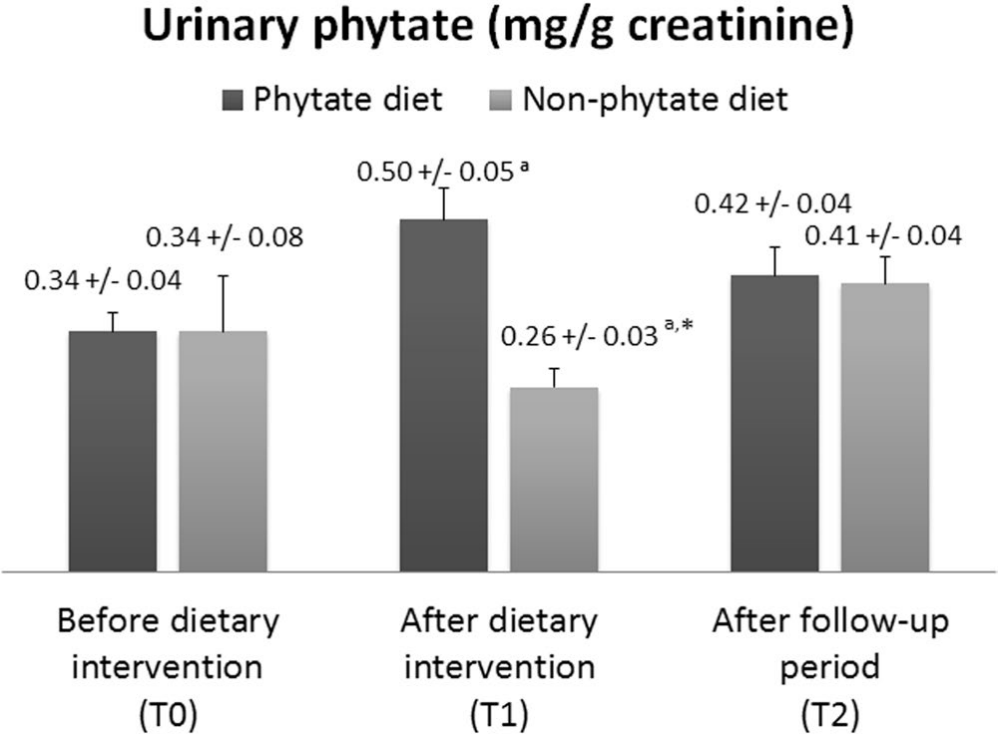
Effect of phytate diet on urinary levels of IPs. Values are expressed as medians ± SEs. Intra-group differences (T_0_ *vs*.T_1_ and T_1_ *vs*. T_2_) were assessed using a paired-sample Wilcoxon signed-rank test or a paired-samples *t*-test (*a*: *p* < 0.05 for T_0_ *vs*. T_1_). Inter-group differences (phytate diet *vs*. non-phytate diet) were assessed using an analysis of covariance after adjusting for baseline levels (*p < 0.05).

At baseline (T_0_), both groups had similar urinary excretion of myo-inositol phosphates (IPs) (Fig. 4). After 3 months (T_1_), patients treated with the IP6 diet had a significant increase in urinary IPs (0.34 ± 0.04 to 0.50 ± 0.05 mg/g creatinine; *p* = 0.005). However, after the follow-up period (T_2_) these patients had a slight but no significant decrease in urinary IPs (0.50 ± 0.05 to 0.42 ± 0.04 mg/g creatinine; *p* = 0.357). Patients who received a non-IP6 diet decreased its urinary IPs at T_1_ (0.34 ± 0.08 to 0.26 ± 0.03 mg/g creatinine; *p* = 0.020), whereas it increased at T_2_ (0.26 ± 0.03 to 0.41 ± 0.04 mg/g creatinine; *p* = 0.478).

### Safety and adverse events

All patients exhibited good tolerance to the IP6 tablets. There were no serious adverse events (death, life-threatening events, or events placing a patient in jeopardy or leading to admission) and no drop-outs related to IP6 supplementation. However, one patient presented had a severe hypoglycemic event while on the non-IP6 diet.

## Discussion

This study is the first to report the effect of IP6 as inhibitor of protein glycation and therefore, on AGEs formation both *in vitro* and in patients suffering of T2DM.

Our *in vitro* studies indicated that IP6 significantly reduced the formation of fluorescent AGEs on Ac-Lys and Ac-Arg (the main protein glycation hot spots) in the presence of Fe^3+^ and ribose, a powerful glycating agent^60^ whose concentration is increased in T2DM patients^61^. This occurs because IP6 is able to strongly chelate Fe^3+^ through the three phosphate groups in positions 1, 2 and 3. These groups are flexible, and bind the Fe^3+^ so that all six coordination sites are occupied by hydroxyl groups, thus yielding a highly stable complex^39^. Once within the IP6-Fe complex, Fe^3+^ notably decreased its ability to catalyze glycation reactions. Accordingly, previous results indicated that IP6 had the same protective effect in blocking the formation of Fe-catalyzed free radicals *via* the Fenton reaction^62^.

The *in vitro* inhibitory effect of IP6 only occurred at prolonged incubation times, thus suggesting that its inhibitory effect mainly affects the post-Amadori reactions, which yields the final formation of AGEs (Fig. 1). Previous studies reported that AGEs inhibitors and AGEs breakers act through chelation of metal cations, either directly or due the action of products of their hydrolysis and/or metabolism^63^. Moreover, several studies have also reported that metal chelators may provide therapeutic benefits for diabetes, cardiovascular disease, and renal disease^63–65^. There is also evidence that some chelators may shift the redox potential of iron and copper, altering their catalytic activity and thereby ameliorating oxidative stress and reducing complications of diabetes. Thus, further consideration should be given to iron and copper chelators as agents for the treatment of oxidative stress and AGEs formation, which lead to major complications in patients with T2DM^63^.

Protein glycation leading to AGEs accumulation are thought to be one of the main triggering factors of the diabetic complications including nephropathy, retinopathy and neuropathy^13–18^. AGEs are not exclusively formed on proteins, but also on other endogenous key molecules such as lipids or nucleic acids. This alters the intracellular signaling and the gene expression, and release pro-inflammatory molecules and free radicals^13–18^. For this reason, natural compounds like IP6 that are able to inhibit protein glycation, can be crucial on the dietary management of type 2 diabetes and its related-complications.

Our *in vitro* results led us to analyze the effect of IP6 on AGEs formation *in vivo*. Thus, we designed a randomized crossover trial of patients with T2DM to investigate the potential therapeutic effects of a 3-month dietary intervention supplemented with IP6. To our knowledge, this is the first study to investigate the effects of IP6 supplementation on AGEs formation in humans with TDM2. Previous studies have used AGEs levels in serum and tissues as a biomarker of aging and longevity^66^, and as a predictor of decreased glomerular filtration rate^67^, heart failure^68^, and mortality in patients undergoing hemodialysis^69^. An increased total AGEs content is also associated with development of kidney disease^70^, obstructive sleep apnea^71^, vascular calcification in patients with kidney disease^72^, and cardiovascular disease in patients on peritoneal dialysis^73^ or with thrombosis^74^. In addition, an elevated AGEs levels may also adversely affect perinatal outcomes in mothers with gestational diabetes^75^. However, the diagnosis of T2DM and quantification of its therapeutic control focuses on the level of HbA1c^76,77^. Therefore, HbA1c is considered a marker of circulating lipids^78^, diabetic retinopathy^79^, nephropathy^80^, and neuropathy^81^, and is used to predict the severity of coronary artery disease^82^ and pathological retinal vasculature (as indicated by fractal dimension)^83^.

Our results indicate that a 3-month IP6 diet significantly lowered the levels of circulating AGEs (~25%) in patients with T2DM. Moreover, these patients also had a 3.8% decline in HbA1c, presumably due to a reduced overall protein glycation^84^. Otherwise, it is difficult to quantify or discard a possible effect of an improvement of the overall glucose metabolism due to the IP6. Although we observed no effect of IP6 intake on fasting glucose and on insulin levels, we did not measure postprandial glucose. Some previous experimental studies suggested that IP6 can decrease intestinal amylase activity, thus diminishing the postprandial glycemic excursion^84^.

AGEs and IP6 have both been linked to inflammation and insulin resistance^85,86^. However, we found that the IP6 diet did not lead to changes in hs-CRP, leukocyte count, or insulin resistance. Nevertheless, we cannot exclude a possible effect of IP6 on other modulators or molecular surrogates of inflammation.

We observed no large changes in body weight during the experimental period. However, there was a minor, but statistically significant, weight gain in patients during the follow-up period (T_2_) after the IP6 diet. This was surprising, and the reasons for this weight gain require further investigation. In diabetic animal models, IP6 supplementation is related to less weight gain and higher levels of leptin^87^. It is possible that withdrawal of IP6 from our patients led to increased caloric intake, together with a decrease in leptin.

We found that the IP6 diet did not affect serum lipid levels and the non-IP6 diet decreased total cholesterol. However, experimental studies reported that sodium IP6 reduces lipase activity, total cholesterol, low density lipoprotein, hepatic total lipids, and hepatic triglycerides, and increases high density lipoprotein^88,89^. In this regard, we should note that we used calcium-magnesium IP6, the naturally occurring form. There is evidence that the calcium-IP6 complex does not bind bile acids. Thus, this could reduce fecal bile excretion and increase cholesterol availability^90^. This data are in accordance with our results, so it was possible that the non-IP6 dietary intervention provoked a decrease in total cholesterol levels by the adherence of the patients to a dietary plan whereas the no lipid changes after IP6-intervention was explained by the modification in fecal bile excretion. In this sense, it would be interesting to investigate the clinical effects of sodium phytate as a dietary supplement.

Moreover, the iron and transferrin levels did not decrease after IP6 intervention. This observation makes IP6 suitable to be considered as a therapy since there is a great deal of controversy regarding the effects of IP6 on the metal absorption. Some studies suggest that the iron absorption might decrease ~3% in diets including low calcium, high phosphorus and high IP6 content^40,41^. However, other studies indicated that a dietary IP6 intake as phytin in a balanced diet (0.1%), had no adverse effects on mineral bioavailability and iron absorption^42–46^. Human trials indicated that phytin intake of 2 g/day did not affect mineral balance^45,46^. Interestingly, the Mediterranean diet results in consumption of approximately 1 g of phytate/day^59^. In any case, our data supports the idea that the dietary phytin intake of 1 g/day, as a part of a balanced diet, had no adverse effects on iron absorption.

There is strong evidence that eating a variety of whole-grain foods and legumes benefits patients with T2DM^91^. In fact, several studies reported improvements in glycemic control and a reduction of HbA1c following increased dietary intake of legumes and whole grains^92,93^. Legumes and whole grains are rich in IP6, so this could explain, at least in part, their beneficial effects. Unfortunately, experimental evidences suggest that in our Mediterranean region, the IP6 consumption is lower in patients with T2DM than in non-diabetic subjects (unpublished data). Based on the results presented here, we strongly suggest that clinicians should actively encourage patients with T2DM to consume a diet rich in IP6.

Although our study is the first to report the effects of IP6 on AGEs *in vitro* and in patients with T2DM, there were several limitations. In particular, we only examined 33 patients, all of whom were from a single medical center. Thus our findings may have limited generalizability. Another limitation is that IP6 supplementation was not blinded. Thus, a large multicenter and blinded placebo-controlled study is needed to strengthen our results.

In conclusion, our results show that IP6, a natural product present legumes and cereals, reduces AGEs formation *in vitro*, and that dietary supplementation with IP6 reduces circulating AGEs and HbA1c levels in patients with T2DM. This protective effect is likely due to IP6-mediated chelation of Fe^3+ ^^94^. Thus we suggest that a diet rich in IP6 could help to prevent complications of diabetes. Furthermore, IP6 consumption may help to prevent or minimize other AGE-related disorders. Large, long-term, and randomized prospective clinical studies must be performed to more completely assess the benefits and risks of IP6 consumption in patients with TDM2.

## Methods

### Reagents

All chemicals were from Sigma-Aldrich Chemical Co. unless otherwise noted.

### *In vitro* studies of AGEs formation

The formation of Fe^3+^-catalyzed AGEs was monitored using fluorescence spectroscopy on a solution containing Ac-Lys, Ac-Arg, ribose, and Fe^3+^ with different concentrations of IP6 (0 to 2 µM). Stock solutions of Ac-Lys, Ac-Arg, D-ribose, FeCl_3_, and IP6 were freshly prepared in 0.2 M phosphate buffer (pH 7.4) that was previously treated with CheleX^TM^ (70 g/L). Stock solutions were then filtered using a 0.22 μm membrane and combined to obtain mixtures containing Ac-Lys (2 mM), Ac-Arg (1 mM), ribose (0.2 M) and 2 µM Fe^3+^. The reaction mixtures and control samples were incubated at 37 °C for 7 days. Aliquots were collected for analysis at different incubation times. Time-dependent AGEs formation was monitored at 25 °C using a Cary Eclipse fluorescence spectrophotometer equipped with a Peltier temperature controlled cell holder (λ_exc_ 320 nm; λ_em_ 360–540 nm). The maximum emission signal was observed at 420 nm and therefore, used to calculate the percent of AGEs inhibition (PAI) after 7 days of incubation:

1 $${\rm{PAI}}=([{I}_{0}-{I}_{I}]/{I}_{0})\cdot 100$$

*I*_0_ is the fluorescence intensity in absence of IP6 and *I*_I_ is the fluorescence intensity in the presence of IP6. These assays were performed in triplicate, and means and standard errors are reported.

### Randomized crossover study of patients with T2DM

#### Subjects and study design

This was a single-center, randomized, crossover, open-label study. The 35 subjects, who were consecutive T2DM patients, were prospectively enrolled from our outpatient clinic at the Endocrinology Department of Hospital Son Llàtzer, a public tertiary care center that covers 250,000 residents of urban and rural areas in the Balearic Islands (Spain).

Volunteers were eligible if they: *(i)* were older than 18 years; *(ii)* had well-controlled T2DM and stable use of medication(s) during the previous 3 months; *(iii)* had HbA1c levels between 6.5–9.0%; and *(iv)* had low dietary consumption of IP6 (estimated from dietary questionnaires). No patient had cancer or clinically significant cardiovascular, liver, or end-stage kidney disease. Two patients did not complete the dietary intervention. Thus, 33 volunteers successfully completed the study. The minimum number of patients for detecting a 20% difference in AGEs level at the end of the study, with 80% power and 95% confidence interval, was 30 patients per group.

Subjects were randomly assigned to the IP6-diet group or to the non-IP6 diet group for 12 weeks. After 12 weeks, each group was given a 12-week washout period, and then switched to the alternate diet for 12 weeks. All patients were then followed for an additional 12 weeks **(**Fig. 2**)**. Patients were asked not change their use of anti-hyperglycemic medications (dose or drug) during the study period, unless they had a fasting blood glucose level above 250 mg/dL during two consecutive measurements or suffered from a severe hypoglycemic event. In addition, changes in the total daily insulin dose greater than 10% were discouraged during the trial.

Randomization sequence was created using Excel software (Microsoft Office 2010) and was stratified with a 1:1 allocation using random block of 4 by an independent researcher with no clinical involvement in the trial. After the clinicians had obtained the patient’s consent, they made contact with the independent researcher of the recruitment process for allocation consignment, enrolling and assessing participants in sequential order. After randomization, there was no blinding of study participants and researchers administering interventions, but there was blinding for researchers assessing outcomes.

#### Dietary intervention

Patients received an IP6 diet (diet plan with IP6 supplementation) or a non-IP6 diet (the same diet plan without IP6 supplementation). The IP6 supplementation consisted of 1 capsule of 380 mg of calcium-magnesium IP6 (Broken Laboratorios, SALVAT S.A.), and was given three times daily with each main meal.

After each intervention, all subjects received oral and written information about their diet plans. The diet plan consisted of a daily meal plan, with five meals per day. Participants were requested to follow the menu plan as much as possible, and to report any meal that differed from what was stipulated.

The diet plan for both groups included fruits, vegetables, fish, shell-fish, meat, eggs, olive oil, and low-fat dairy products. Participants in both groups were specifically advised to avoid foods rich in IP6, such as legumes, cereals, and nuts. In these cases, the main sources of carbohydrates were white bread, white rice (not basmati), pasta, white flour, potatoes and non-whole wheat semolina. This diet is in accordance with the dietary recommendations of Spain for people with T2DM, regarding macronutrient composition, dietary fiber, minerals, and vitamins.

A dietician followed each patient monthly to ensure that the dietary plan was followed. The dietician also checked for compliance (consumption of at least 80% of pills) at every visit by counting of pills.

#### Variable Outcomes

The main outcome measure was serum level of AGEs. The secondary outcomes were HbAc1, fasting glucose, insulin, homeostatic model assessment (HOMA) of insulin resistance, lipids, blood pressure, and weight. Changes in C-reactive protein (CRP) and leukocyte count, which are related to inflammation, were also analyzed.

Clinical histories were extracted from the electronic medical records. Furthermore, data from anamnesis, laboratory analysis, and physical examinations were prospectively collected during the trial. Physical and anthropometric measurements were carried out by qualified personnel while the subjects were barefoot and wearing light clothes. Urine and blood samples were taken before and after the dietary interventions, and after the follow-up period. Blood samples were collected in the morning (after 12 h of fasting). These samples were left to stand for 30 min at room temperature, and the serum was then separated by centrifugation. Hematimetric and biochemical analyses were performed in an automated analyzer (Cell-Dyn Sapphire and Architect ci16200, Abbott). Insulin was analyzed by chemiluminescent-immunometric assay (Advia Centaur, Siemens). Highly sensitive CRP (hs-CRP) and lipoprotein (a) (Lp [a]), were analyzed by Nephelometry (Immage 8000, Beckman Coulter). All samples were run in duplicate, and the coefficients of intra- and inter-assay variation were below 10%.

Blood pressure was measured 3 times consecutively after 5 min of rest while the subject was sitting quietly. The average of the second and third measurements was recorded. Patients using anti-hypertensive drugs and those with systolic blood pressure of 140 mmHg or more and/or diastolic blood pressure of 90 mmHg or more were categorized as having hypertension^95^.

Dyslipidemia was defined as the presence of one of the following: LDL cholesterol levels of 100 mg/dL or more, HDL cholesterol below 40 mg/dL (men) or 50 mg/dL (women), triglycerides of 150 mg/dL or more, or use of a lipid-lowering drug^96^.

#### Serum AGEs determination

AGEs in serum samples were measured using the Cell Biolabs’ OxiSelect™ AGEs Competitive ELISA Kit, which provides a rapid detection and quantification of circulating AGEs. Quantitation was determined by using an AGE-BSA standard curve.

#### Estimation of IP6 consumption

IP6 consumption was estimated as previously described^51,59^. Briefly, it was based on a Food Frequency Questionnaire (FFQ) which considered 10 items that were major sources of IP6 (whole cereals, legumes, and nuts), the serving size of each item, and the IP6 concentration of each item. The same day, a 2-h urine sample was collected, and the dietician interviewed each patient to confirm the answers to the 10 selected FFQ items. IP6 consumption was determined by consumption of foods in 3 major food groups: legumes, whole grains, and nuts. Low IP6 consumption was defined by the consumption of these foods fewer than three times a week. Previous studies from our laboratory indicated a good correlation between declared IP6 consumption and IP6 levels in urine and other tissues^97,98^. These studies indicated that consumption of foods rich in IP6 at least three times a week is sufficient to achieve a high serum level of IP6.

#### Measurement of urinary IPs

Urinary IPs was measured at 2 h after the first urine of the morning. For this test, 20 mL of fresh urine was acidified with HCl (1:1) to pH 3, and then diluted with 20 mL of milli-Q water. This solution was transferred to a 100 mL beaker containing 0.5 g of AG1-X8 resin (anion exchange resin), without previous conditioning. This mixture was stirred with an orbital stirrer at 160 rpm for 15 min. The resin and urine were transferred into a 20 mL solid phase extraction (SPE) tube with a frit, and urine was passed through to separate it from the resin. The resin was then washed with 120 mL of 50 mM HCl and 2 × 5 mL of deionized water. Finally, IP6 was eluted by 4 × 1 mL of 2 M NaCl, with contact between the resin and each 1 mL portion maintained for 5 min by mixing with an orbital stirrer (180 rpm). The final 4 mL of eluate were collected into a single tube, and the solution was mixed prior to IP6 quantification. IPs were determined by indirect phytate analysis of this eluate, using the aluminum-pyrocatechol violet (Al-PCV) system^99^. The two reagents (RI and RII) were prepared daily. RI was a mixture of 0.6 mL of 4 mM Al[NO_3_]_3_ and 4.4 mL of 1.5 M acetic acid/acetate buffer at pH 5.2; RII was a mixture of 0.6 mL of 5.6 mM PCV and 4.4 mL of deionized water. IP6 standards in the range 1–10 µM were prepared in 2 M NaCl. The assays were performed in 96-well plates, with each well containing 30 µL RI, 290 µL of a standard or eluate, and 30 µL of RII. After incubation for 15 min, absorbance was measured at 570 nm. All samples were assayed in duplicate. This method does not discriminate between IP6 and other IPs, so the measured parameter is considered to be ‘phytic acid equivalents’. We carried out the determination of the urinary IP6 to have an analytical parameter to confirm the adherence of patients to the dietary interventions.

### Statistical analysis

Data are presented as means (standard deviations), medians (interquartile ranges), or numbers (percentages). Intergroup comparisons at baseline (T_0_, before intervention) were analyzed using the independent-samples *t*-test or the Mann-Whitney U test for continuous variables, and the chi-square test or Fisher’s exact test for categorical variables. Intragroup differences (before [T_0_] *vs*. after intervention [T_1_]; after intervention [T1] *vs*. after follow-up [T_2_]) were evaluated using a paired samples *t*-test or Wilcoxon’s signed-rank paired test for continuous variables, and the McNemar test for dichotomized variables. Intergroup comparisons (after the intervention and after the follow-up period) were assessed using analysis of covariance and Fisher’s exact test, with adjustment for changes in categorical and continuous variables according to baseline values. Bivariate associations were evaluated by Pearson’s or Spearman’s correlation coefficients.

A two-tailed *p*-value less than 0.05 was considered statistically significant. Statistical analyses were performed using SPSS version 23.0 (SPSS Inc., Chicago, IL, USA).

### Ethical considerations

The study design was approved by the Research Committee of Hospital Son Llàtzer and Research Ethics Committee of Balearic Islands [CEI-IB] (IB1933/12 PI). The clinical trial number is EudraCT n° 2017-003609-16, which was validated on 22/02/2018. The Universal Trial Number (UTN) is U1111-1201-5736. All patients provided written informed consent before participation. All experiments were performed in accordance with relevant guidelines and regulations.
